# Brownfield redevelopment evaluation based on structure-process-outcome theory and continuous ordered weighted averaging operator-topology method

**DOI:** 10.1038/s41598-023-44793-1

**Published:** 2023-10-16

**Authors:** He Jian, Hu Hao, Jiang Haidan, Pan Haize, Liu Chuan

**Affiliations:** 1 College of Civil Engineering, Chongqing University of Arts and Sciences, Chongqing, 402160 China; 2 School of Civil Engineering and Geomatics, Southwest Petroleum University, Chengdu, 610500 China

**Keywords:** Environmental sciences, Environmental social sciences, Engineering, Mathematics and computing

## Abstract

As an important part of urban renewal, brownfield restoration and renovation are of great significance to the sustainable development of cities. The structure-process-outcome theory was introduced into this study to improve the rationality and scientific vigor of the redevelopment assessment process and to evaluate whether brownfield sites meet the conditions for redevelopment. Based on this theory, the relationship among structures, processes and outcomes can be well elucidated. Specifically, a good structure should contribute to an effective process, which will increase the possibility of a favorable outcome. The basic conditions, practice principles, and result orientation in the whole procedure of brownfield redevelopment were comprehensively analyzed. In addition, a more complete and reasonable three-level evaluation index system for brownfield redevelopment was established. In order to reduce the subjectivity in the evaluation process, an unbiased scientific brownfield redevelopment evaluation model was constructed using the continuous ordered weighted averaging operator-topology method. The evaluation decision system was applied to the renovation of a tract project in Chengdu, China. The results proved that the model could effectively and accurately evaluate the quality level of the brownfield redevelopment project, and the proposed recommendations can provide a basis for decision-making.

## Introduction

The term “brownfield” was widely used in Western countries after being proposed at a hearing in the U.S. Congress in June 1992. According to the United States Environmental Protection Agency (USEPA), brownfields are defined as lands that are developed, partially or fully utilized, as well as those that are unused, potentially contaminated, and difficult to redevelop^1^.

With the ongoing urbanization and industrialization of China, the scale of cities and urban population has increased dramatically. The rapidly rising demand for urban construction land has greatly intensified the pressure on land supply. During the 40 years of reform and opening up, the urbanization rate of China rose rapidly from 17.92% in 1978 to 64.7% in 2021, and the urban land demand increased 4.72 times from 12,856 km^2^ in 1990 to 60,721 km^2^ in 2020^2^. However, with the closure and relocation of historical industrial areas, the number of brownfields has shown a trend of yearly increase, and urban wasteland is also on the upswing. In 2012, China reported approximately 300,000 brownfields covering a total area of about 20 million hm^2^^3^. In 2014, there were about 4.2 million brownfield sites in the EU, 340,000 of which were at risk of contamination^4^. In 2018, there were over 450,000 estimated developable brownfield sites in the United States. In 2020, there were 21,000 estimated brownfield sites in England, which could transform into 1.06 million households after redevelopment^5^. The emergence of brownfields is indicative of land resource waste, causes potential environmental pollution and leads to the decline of urban life quality. Therefore, in order to realize the full utilization of urban land resources and improve the urban living environment, the restoration and redevelopment of brownfield is imperative.

In order to fully utilize the construction land and protect the environment, the Chinese Ministry of Land and Resources issued *the Regulations on the Economical and Intensive Use of Land* in May 2014^6^. In November 2016, the Ministry of Land and Resources issued *the Guiding Opinions on Further Promoting the Redevelopment of Low-Use Land in Urban Areas*^7^. The focus on urban land use has gradually shifted from “incremental” to “stock”. In December 2016, the Chinese Ministry of Environmental Protection promulgated *the Measures for Soil Environmental Management of Contaminated Land*^8^. In 2017, Chinese provinces began to publish a list of risk control measures for contaminated land, and the management and redevelopment of brownfields began to receive attention^9^. In 2020, Chinese President Xi Jinping delivered an important speech at the 75th General Debate of the United Nations General Assembly, setting out the goal and vision for China to strive to reach peak carbon emissions by 2030 and achieve carbon neutrality by 2060. Green development has become an inherent requirement for high-quality urban development. However, there are a series of obstacles to redeveloping and utilizing brownfield sites. It is also difficult to understand the economic benefits of redeveloping brownfield sites due to their superior location, pollution issues, high costs, and the involvement of numerous stakeholders.

This paper aims at the ex-ante evaluation of brownfield redevelopment and the assessment of whether brownfield sites meet the conditions for redevelopment. It also focuses on addressing the issues concerning the rationality and scientific vigor of the redevelopment assessment process. In the second part of this paper, we reviewed the research on brownfield development worldwide and pointed out the research gap. In the third section, a brownfield redevelopment evaluation index system was developed based on the structure-process-outcome theory. In the fourth part, a brownfield redevelopment evaluation model was constructed based on the continuous ordered weighted averaging (C-OWA) operator-topology method, and the fifth part is a case analysis, which verifies the applicability of the evaluation indexes and the evaluation model. The evaluation indexes and evaluation models proposed in this study are more comprehensive, reliable and applicable and provide a good basis for the decision-making related to brownfield redevelopment.

## Literature review

As an important land section in urban development, the economic and social benefits of brownfields have received extensive attention. In 2006, Shen^10^ underscored the importance of the reuse of “brownfields” in the transformation of resource-based cities. By combining brownfields with the construction of urban green space networks, new impetus can be generated for sustainable urban development. In 2010, Adams^11^ stated that brownfields should be considered as a development opportunity rather than a planning issue. In 2017, Ni^12^ pointed out that brownfield regeneration is an important way for sustainable urban development and an important source of urban green open space. In 2018, Naveed^13^ stated that the identification and assessment of brownfields are necessary to address environmental issues and promote sustainable development. Based on a survey of 200 brownfield sites in the United States from 2000 to 2015, Green^14^ concluded that socioeconomic factors (income levels), green development, and tax incentives are significantly associated with brownfield redevelopment. In 2019, Zhao and Zang^15^ pointed out that the location of brownfield sites significantly influences investors’ decisions and brownfield regeneration in the urban renewal process. In 2020, Zhong et al.^16^ proposed a conceptual framework for ex-ante evaluation of brownfield greening based on ecosystem services assessment, economic cost–benefit analysis, and spatial pattern analysis. Based on a survey of brownfield redevelopment decision systems over the past 20 years, Hammond et al.^17^ argued that these decision systems fall short in addressing the complexity of brownfields from a sustainability perspective. The above studies illustrate that brownfield redevelopment has a significant impact on developing high-quality, green, and sustainable cities. However, it is difficult to determine whether a brownfield meets the development standards because of the possibility of contamination and the complicated surrounding environment. Therefore, a thorough pre-evaluation is crucial to guarantee the effectiveness of the redevelopment project. Accordingly, this paper introduces the structure-process-outcome theory to the assessment of brownfield redevelopment. Based on this theory, the relationship among structure, process, and outcome can be clearly elucidated. Specifically, a good structure should promote an effective process, and an effective process will increase the likelihood of an ideal outcome. In addition, a more thorough evaluation index system from the dimensions of structure, process, and outcome can be established. In order to reduce the subjectivity in the evaluation process, the C-OWA operator-topology method is applied to establish the brownfield redevelopment evaluation model. Its applicability, scientific vigor, and rationality are verified in practical cases. The results show that the evaluation system provides a good basis for decision-making on brownfield redevelopment.

## Establishment of Brownfield redevelopment evaluation index system

### Structure-process-outcome theory

In 1966, Donabedian argued that the quality of medical care services should be understood as the expectation that comprehensive and rational medical services can fulfill the physical and mental health goals of patients^18–20^. In addition, the classical quality evaluation model of structure-process-outcome was proposed, which aimed to evaluate healthcare service quality with an emphasis on context, actions and effects. Structure refers to the original basic conditions in the organization, including hardware and software facilities for service delivery, human and material input, and other objective resources. Process refers to the services provided to clients in a specific process, including physical and psychological care, training of staff, and monitoring of the process and other implementation measures. The outcome is the desired state after receiving the service. It is also the feedback on the structure and process, such as the physical and mental changes and service satisfaction. “Structure-process-outcome” is a holistic system with complex interrelationships that do not present a simple linear pattern. The structure is static, and the process is dynamic. The quality of structure and process inevitably affects the outcome, with the process exerting a more direct impact. Therefore, good structure and process can positively contribute to the outcome^21–24^.

Based on the three dimensions, namely, “structure, process, and outcome”, the whole procedure of the brownfield development process is analyzed. The influencing factors are studied, and the information elements are examined to form a more scientific and comprehensive evaluation index system for brownfield redevelopment^25,26^.

### Metrics construction process

The structural dimension measures all physical infrastructure conditions related to brownfield sites in the redevelopment process^27^. In terms of brownfield conditions, the degree and type of pollution affect the difficulty of environmental remediation. The location, size, and traffic conditions of brownfield sites impact the value of investments. Moreover, human factors such as literacy, income level, and the relationship between neighbors also determine the difficulty of the redevelopment process. Therefore, accurate information on the structural dimensions of brownfields is the basis for redevelopment management.

The process dimension mainly refers to practical principles, human decision-making management, and organizational coordination of the brownfield redevelopment process. In the process of brownfield redevelopment, capital investment (e.g., environmental remediation and renovation construction costs), matching measures (e.g., regional planning, government policy support and supervision, and management), basic conditions of construction and renovation enterprises, and public opinion guidance (e.g., access to environmental information) significantly affect the process of brownfield redevelopment. Therefore, coordinating the relevant interests of all participants is the key to the success of the project^28^.

There are two main sources of funding for project implementation: the government and social capital. The outcome orientation is the main factor considered by investors. Therefore, the outcome dimension mainly involves measuring the economic and social benefits of brownfield redevelopment projects. Economic benefits include expected revenue and the length of the payback period, and the social benefits are the satisfaction of the surrounding residents and the added value of the surrounding area^29^.

Based on the three dimensions, relevant studies, and expert consultation, the evaluation index system for brownfield redevelopment is established, as shown in Table 1.

**Table 1:** Brownfield redevelopment evaluation index system based on the “structure-process-outcome” theory.

Primary index	Secondary index	Three-level index
Structure (S_1_)	Geographical Environment (S_11_)	Brownfield Location (S_111_)
		Area size (S_112_)
		Usage Status (S_113_)
		Traffic conditions (S_114_)
	Human conditions (S_12_)	Neighborhood Avoidance (S_121_)
		Income level of residents (S_122_)
		Environmental awareness of brownfield (S_123_)
		Residents’ willingness to renovate (S_124_)
	Contamination Status (S_13_)	Pollution category (S_131_)
		degree of pollution (S_132_)
		The difficulty of land restoration (S_133_)
Process (P_2_)	Capital investment (P_21_)	Construction Costs (P_211_)
		pollution treatment costs (P_212_)
		Financing Capacity (P_213_)
		Funding Matching (P_214_)
	Preferential policy (P_22_)	Regional Planning (P_221_)
		Special support policy (P_222_)
		Management organization (P_223_)
		Regulatory Measures (P_224_)
	Technology Assurance (P_23_)	Number of experts (P_231_)
		Construction enterprise qualification (P_232_)
	Information Orientation (P_24_)	Public opinion guidance (P_241_)
		Information Disclosure (P_242_)
Outcome (O_3_)	Investment income (O_31_)	Expected return (O_311_)
		Payback period (O_312_)
	Social benefits (O_32_)	Resident satisfaction (O_321_)
		Regional Value-Added (O_322_)

## C-OWA operator-topological evaluation model

### Calculation of index weights of C-OWA operator

Due to excessive evaluation indexes, cross-influence of indexes, different expert perceptions, and personal preferences, the disturbance of subjective emotional factors in weight assignment by the traditional hierarchical analysis method, entropy method, or factor analysis method is unavoidable, significantly affecting scientific validity^30^. Due to the characteristic resembling a normal distribution and the close relevance to the decision data, the C-OWA operator method proposed by Professor Yager in 2004 is widely used in decision-making management. With this method, the subjectivity of the weight calculation can be avoided, and the fairness of the decision-making can be enhanced. The C-OWA operator weighting method can contribute to a rational and scientifically robust evaluation of brownfield redevelopment and solve the intricacies arising from the involvement of a large number of evaluation indexes, the need for modeling, and the uncertainty in the index data^31^.

### Establishing the initial evaluation matrix

Experts were invited to score by the Delphi method on a scale of 1–10. According to relevant studies^32–34^, the importance scores of brownfield redevelopment evaluation indexes were divided into five levels, with [9,10) representing level I (extremely important), [8,9) indicating level II (very important), [7,8) representing level III (quite important), [6,7) representing level IV (somewhat important), and [0,6] indicating level V (not important). The initial evaluation matrix $A\left( {a_{1} ,a_{2} , \ldots ,a_{n} } \right)$ was obtained and then sorted in the order of scores from largest to smallest to yield $B\left( {b_{0} ,b_{1} , \ldots ,b_{n - 1} } \right)$, where $b_{0} > b_{1} > b_{2} > \cdots > b_{n - 1}$.

### Calculation of the weighted vector

The weighted vector $\chi_{j + 1}$ about the vector $B$ is calculated through the following equation:

1 $$\chi_{j + 1} = \frac{{C_{m - 1}^{j} }}{{\sum\limits_{j = 0}^{m - 1} {C_{m - 1}^{j} } }} = \frac{{C_{m - 1}^{j} }}{2m - 1}$$

where $\sum\nolimits_{j = 0}^{m - 1} {\chi_{j + 1} } = 1$ and $j = 0,\;1,\;2 \ldots ,m - 1$; $m$ is the number of experts.

### Calculation of the absolute weight

The absolute weight $\varpi_{i}$ of each index factor $i$ is calculated by the weighted vector $\chi_{j + 1}$:

2 $$\varpi_{i} = \sum\limits_{j = 0}^{m - 1} {\chi_{j + 1} b_{j} } ,\;i = 1,2, \ldots ,n$$

where $i = 0,1,2 \ldots ,n$; $n$ is the number of indexes.

### Calculation of relative weight

The relative weight $\omega_{i}$ of each index factor $i$ is calculated by the absolute weight $\varpi_{i}$:

3 $$\omega_{i} = \frac{{\varpi_{i} }}{{\sum\limits_{i = 1}^{n} {\varpi_{i} } }}$$

## Topologically integrated evaluation model

Topologism was proposed by Wen^35^ in 1983. It is an innovative method to explore expansion with a formal model for solving contradiction. Currently, topology is widely used in many fields, such as engineering, management, economics, and philosophy, and it combines qualitative and quantitative approaches^36^. The introduction of the comprehensive evaluation model based on the concept of topology will facilitate the hierarchical representation of the quality level of brownfield redevelopment, clarify the evaluation level of each index factor in a formal and organized manner, and guide the decision-making related to brownfield redevelopment, the matters that should be paid attention to, and the corresponding countermeasures.

### Determination of the classical domain, the section domain, and object elements

According to the topological theory, the characteristics, quantitative values, objects, and changes of the contradictory problems of brownfield redevelopment quality evaluation can be expressed based on the topological distance. The primitives (characteristics, quantitative values, objects) are established to classify the brownfield redevelopment quality evaluation into classical and nodal domains^37^.

1. A certain level of rating index corresponding to the quality evaluation of brownfield redevelopment and the value interval is considered the classical domain $R_{i}$, which can be expressed as follows:
  4 $${R_i} = ({N_i},{C_j},{V_{ij}}) = \left[ {\begin{array}{*{20}{c}} {{N_i}}&{{c_1}}&{\left\langle {{a_{i1}},{b_{i1}}} \right\rangle }\\ {}&{{c_2}}&{\left\langle {{a_{i2}},{b_{i2}}} \right\rangle }\\ {}&{\vdots}&{\vdots}\\ {}&{{c_m}}&{\left\langle {{a_{im}},{b_{im}}} \right\rangle } \end{array}} \right]$$
  where $N_{i}$ is the $i$th evaluation level of brownfield redevelopment quality evaluation; $C_{j}$ is the evaluation index; $V_{ij}$ is the value range of the corresponding evaluation index.
2. The nodal domain $R_{P}$ is determined with the following equation:
  5 $${R_p} = ({N_p},{C_j},{V_{pj}}) = \left[ {\begin{array}{*{20}{c}} {{N_p}}&{{c_1}}&{\left\langle {{a_{p1}},{b_{p1}}} \right\rangle }\\ {}&{{c_2}}&{\left\langle {{a_{p2}},{b_{p2}}} \right\rangle }\\ {}&{\vdots}&{\vdots}\\ {}&{{c_m}}&{\left\langle {{a_{pm}},{b_{pm}}} \right\rangle } \end{array}} \right]$$
  where $N_{p}$ is all grades of brownfield redevelopment quality evaluation; $C_{j}$ is the evaluation index; $V_{pj}$ is the value range of the corresponding evaluation index.
3. The primitive $R_{x}$ is determined with the following equation:
  6 $$R_{x} = (P,C_{j} ,V_{j} ) = \left[ {\begin{array}{*{20}c} {\begin{array}{*{20}c} {N_{p} } & {c_{1} } & {\nu_{1} } \\ \end{array} } \\ {\begin{array}{*{20}c} {} & {c_{2} } & {\nu_{2} } \\ \end{array} } \\ {\begin{array}{*{20}c} {\begin{array}{*{20}c} {} & {} \\ \end{array} } & \vdots & \vdots \\ \end{array} } \\ {\begin{array}{*{20}c} {\begin{array}{*{20}c} {} & {} \\ \end{array} } & {c_{m} } & {\nu_{m} } \\ \end{array} } \\ \end{array} } \right]$$
  where $P$ is the unit number; $C_{j}$ is the evaluation index; $V_{j}$ is the value of the corresponding evaluation index.

### Determination of the association function

1. Calculation of the topologizable distance. Based on the classical domain and the nodal domain, the topologizable distance between each evaluation index of the primitive and the classical domain and the nodal domain can be calculated as follows:
  7 $$\begin{aligned} \rho \left( {\nu_{i} ,V_{ij} } \right) & = \left| {\nu_{i} - \frac{{a_{ji} + b_{ji} }}{2}} \right| - \frac{1}{2}\left( {b_{ji} - a_{ji} } \right) \\ \rho \left( {\nu_{i} ,V_{pj} } \right) & = \left| {\nu_{i} - \frac{{a_{pi} + b_{pi} }}{2}} \right| - \frac{1}{2}\left( {b_{pi} - a_{pi} } \right) \\ \end{aligned}$$
2. Calculation of correlation degree. The correlation degree reflects the correlation between the evaluation indexes and each evaluation level, which can be expressed as follows:
  8 $$\begin{gathered} K_{i} \left( {V_{i} } \right) = \left\{ {\begin{array}{*{20}c} {\frac{{\rho \left( {\nu_{i} ,V_{ij} } \right)}}{{\rho \left( {\nu_{i} ,V_{pj} } \right) - \rho \left( {\nu_{i} ,V_{ij} } \right)}}\begin{array}{*{20}c} {} & {\begin{array}{*{20}c} {} & {\nu_{i} \in V_{ij} } \\ \end{array} } \\ \end{array} } \\ {\frac{{ - \rho \left( {\nu_{i} ,V_{ij} } \right)}}{{\left| {V_{ij} } \right|}}\begin{array}{*{20}c} {} & {\begin{array}{*{20}c} {\begin{array}{*{20}c} {\begin{array}{*{20}c} {\begin{array}{*{20}c} {} & {} \\ \end{array} } & {} \\ \end{array} } & {} \\ \end{array} } & {\nu_{i} \notin V_{ij} } \\ \end{array} } \\ \end{array} } \\ \end{array} } \right. \hfill \\ \hfill \\ \end{gathered}$$
3. Calculation of the total correlation degree. Based on the value $K_{ti} (V_{ij} )$ of the correlation degree of each index evaluation level $i$ and the corresponding weight value $\omega_{j}$, the total correlation degree $K_{j} (N_{j} )$ can be obtained:
  9 $$K_{j} (N_{j} ) = \sum {\omega_{j} } K_{i} \left( {V_{j} } \right)$$

According to the principle of maximum membership $K_{j0} (N_{j} ) = \mathop {\max }\nolimits_{{j \in \left( {1,2, \cdots ,m} \right)}} K_{j} (N_{j} )$, the grade of the redevelopment quality of the brownfield can be obtained^38^.

## Case study

### Project overview

Sanxiang Reconstruction Project is located at the junction of the old and new city on Guihu East Road, Xindu District, Chengdu City, Sichuan Province, China. The area is well-equipped with infrastructure and medical facilities. It belongs to the core of the city, with convenient transportation, developed businesses and abundant educational resources. The area was built in the 1980s, mainly for residence, involving nine old neighborhoods. Most houses are resettlement houses, and some are small factories and workshops. Over the years, the area has suffered from outdated facilities, backward management, poor sanitation, lack of service support and poor overall appearance due to local characteristics, such as dense buildings and population, narrow roads and space, and mixed pedestrian and vehicles. In addition, some of the building entrances are dilapidated, and the terrain is prone to waterlogging. Fitness and recreational facilities for residents are outdated and missing, and some owners occupy public space for private construction. These problems have seriously affected the normal life of residents.

In recent years, the government began to vigorously renovate old urban courtyards to reasonably use urban space and prevent and resolve major security risks. It has also focused on improving the living environment, making up for functional shortcomings, and improving governance services, thus creating a harmonious community with a more beautiful environment, complete functions, and convenient living. Since 2023, the area has been included in the list of renovation and upgrading.

### Calculation of index weights by C-OWA operator

To evaluate the feasibility of redeveloping this brownfield site, influencing factors were scored (1 to 10) according to the established evaluation index system, with higher scores indicating greater importance. Six experts from different backgrounds, including government, environmental, construction, development, academic and consulting sectors, were invited to score the importance of the indexes. The final scores obtained are shown in Tables 2, 3 and 4.

**Table 2:** Expert score of primary indexes.

Primary index	Expert 1	Expert 2	Expert 3	Expert 4	Expert 5	Expert 6
S_1_	9.0	8.5	8.0	8.0	7.8	8.6
P_2_	9.0	9.5	8.5	9.0	9.3	8.0
O_3_	8.5	9.5	8.3	8.9	9.0	9.3

**Table 3:** Expert score of secondary indexes.

Secondary index	Expert 1	Expert 2	Expert 3	Expert 4	Expert 5	Expert 6
S_11_	9.30	8.60	8.70	8.90	8.60	9.60
S_12_	7.50	7.80	8.30	6.70	8.20	6.80
S_13_	8.50	8.60	9.00	8.90	9.20	8.50
P_21_	9.00	8.50	8.00	8.00	8.70	9.00
P_22_	9.00	9.20	8.70	8.00	7.50	8.50
P_23_	8.50	8.00	8.90	7.80	8.50	8.40
P_24_	7.00	7.80	8.00	7.80	7.90	7.60
O_31_	8.20	8.50	8.70	9.00	8.80	9.30
O_32_	9.00	9.50	9.00	8.50	8.40	8.70

**Table 4:** Expert score of three-level indexes.

Three-level index	Expert 1	Expert 2	Expert 3	Expert 4	Expert 5	Expert 6
S_111_	9.20	9.40	9.80	9.50	9.90	10.00
S_112_	8.30	7.90	9.00	8.90	7.10	7.20
S_113_	8.40	8.50	8.50	7.30	7.70	7.40
S_114_	8.00	8.60	8.40	9.20	8.70	8.60
S_121_	7.10	7.20	8.60	7.30	8.70	8.60
S_122_	5.40	6.00	4.80	6.10	7.00	6.70
S_123_	8.00	8.50	8.60	8.70	7.80	9.00
S_124_	8.00	8.70	8.80	7.90	9.40	8.30
S_131_	8.00	7.80	8.60	8.50	7.20	6.70
S_132_	8.20	8.00	8.70	7.70	9.00	7.90
S_133_	8.00	8.50	8.40	8.30	8.10	7.80
P_211_	8.00	8.60	7.70	7.80	8.30	8.00
P_212_	7.50	7.90	8.30	8.40	8.20	8.40
P_213_	8.00	9.00	9.40	9.30	8.50	8.40
P_214_	8.20	6.00	7.80	7.40	7.80	8.30
P_221_	9.20	8.30	9.00	8.40	8.80	8.70
P_222_	8.40	7.80	7.90	9.00	8.70	9.20
P_223_	9.40	9.30	8.70	8.60	9.50	8.70
P_224_	7.80	7.90	8.00	8.60	8.70	8.50
P_231_	8.50	8.70	8.50	8.70	9.00	9.20
P_232_	7.90	7.60	8.10	7.60	8.70	7.80
P_241_	7.80	8.20	7.80	7.90	8.70	8.40
P_242_	7.80	8.80	7.60	8.20	8.30	8.50
O_311_	8.40	8.50	8.70	8.80	9.20	9.10
O_312_	8.40	7.80	8.00	8.60	8.30	7.90
O_321_	9.60	9.50	9.30	9.00	9.10	8.70
O_322_	9.00	9.30	8.50	9.60	8.00	7.90

### Calculation of primary index weights

The expert scores of the primary indexes were obtained, as shown in Table 2.

The weights were calculated using structural indexes as an example, and the score results were reordered from largest to smallest:

$$B = \rm {(}{9}{\text{.0,}}\;{8}{\text{.6,}}\;{8}{\text{.5,}}\;{8}{\text{.0,}}\;{8}{\text{.0,}}\;{7}{\text{.8)}}$$

The weight of each data was calculated according to Eq. (1):

$$\chi_{6} = \rm {(}{0}{\text{.0313,}}\;{0}{\text{.1563,}}\;{0}{\text{.3125,}}\;{0}{\text{.3125,}}\;{0}{\text{.1563,}}\;{0}{\text{.0313}})$$

The absolute weights of indexes were calculated by Eq. (2):

$$\varpi = ({8}{\text{.2750}},\;{ 8}{\text{.9531,}}\;{8}{\text{.9313}})$$

The relative weight of each index was obtained by Eq. (3):

$$W = ({0}{\text{.3163,}}\;{0}{\text{.3423,}}\;{0}{\text{.3414 )}}$$

### Calculation of secondary index weights

The expert scores of the secondary indexes were obtained, as shown in Table 3.

The calculation of absolute weights is consistent with that of the primary index weights, and the results are as follows:

$$\varpi_{1} = ({8}{\text{.8656}},\;{7}{\text{.5938}},\;{8}{\text{.7563)}}$$

$$\varpi_{2} = ({8}{\text{.5625,}}\;{ 8}{\text{.5531,}}\;{8}{\text{.3813,}}\;{7}{\text{.7656)}}$$

$$\varpi_{3} = ({8}{\text{.7500,}}\;{8}{\text{.8250)}}$$

The relative weights of the indexes are:

$$W_{1} = ({0}{\text{.3516,}}\;{ 0}{\text{.3012,}}\;{ 0}{\text{.3472 )}}$$

$$W_{2} = ({0}{\text{.2574,}}\;{ 0}{\text{.2571,}}\;{0}{\text{.2520,}}\;{0}{\text{.2335)}}$$

$$W_{3} = ({0}{\text{.4979,}}\;{ 0}{\text{.5021)}}$$

### Calculation of three-level index weights

The expert scores of the three-level indexes were obtained, as shown in Table 4.

Similar to the first two levels, the absolute weight can be obtained as follows:

$$\varpi_{11} = ({9}{\text{.6469,}}\;{8}{\text{.0813,}}\;{8}{\text{.0094 ,}}\;{8}{\text{.5844}})$$

$$\varpi_{12} = ({7}{\text{.9313 ,}}\;{6}{\text{.0406,}}\;{ 8}{\text{.4781,}}\;{8}{\text{.4781}})$$

$$\varpi_{13} = ({7}{\text{.8688,}}\;{8}{\text{.1781,}}\;{8}{\text{.1969}})$$

$$\varpi_{21} = ({8}{\text{.0250,}}\;{8}{\text{.2000,}}\;{8}{\text{.7781,}}\;{7}{\text{.7594}})$$

$$\varpi_{22} = ({8}{\text{.7344,}}\;{8}{\text{.5156,}}\;{9}{\text{.0188,}}\;{ 8}{\text{.2500}})$$

$$\varpi_{23} = ({8}{\text{.7250,}}\;{ 7}{\text{.8688}});\;\;\varpi_{24} = ({8}{\text{.0781,}}\;{8}{\text{.2156}})$$

$$\varpi_{31} = ({8}{\text{.7688,}}\;{8}{\text{.1531);}}\;\;\varpi_{32} = ({9}{\text{.2125,}}\;{8}{\text{.7188}})$$

The relative weights of the indexes are:

$$W_{11} = ({0}{\text{.2811}},\;{ 0}{\text{.2355}},\;{ 0}{\text{.2333}},\;{0}{\text{.2501}})$$

$$W_{12} = ({0}{\text{.2564}},\;{0}{\text{.1954}},\;{0}{\text{.2741}},\;{0}{\text{.2741}})$$

$$W_{13} = ({0}{\text{.3246}},\;{ 0}{\text{.3373}},\;{0}{\text{.3381}})$$

$$W_{21} = ({0}{\text{.2449}},\;{0}{\text{.2504}},\;{0}{\text{.2679}},\;{ 0}{\text{.2368}})$$

$$W_{22} = ({0}{\text{.2530}},\;{0}{\text{.2467}},\;{ 0}{\text{.2613}},\;{0}{\text{.2390}})$$

$$W_{23} = ({0}{\text{.5258}},\;{ 0}{\text{.4742}});\;\;W_{24} = ({0}{\text{.4958}},\;{ 0}{\text{.5042}})$$

$$W_{31} = ({0}{\text{.5182}},\;{0}{\text{.4818}});\;\;W_{32} = ({0}{\text{.5138}},\;{0}{\text{.4862}})$$

## Quality evaluation of brownfield redevelopment based on the topology method

### Element determination of classical domain, section domain and object to be evaluated

1. Each three-level index S_111_, S_112_, …, O_311_, O_312_ were assigned as $c_{1} \sim c_{27}$, and the classical domain of level I is:
  $$R_{1} = \left[ {\begin{array}{*{20}c} {\begin{array}{*{20}c} {O_{1} } & {c_{1} } & {9,10} \\ \end{array} } \\ {\begin{array}{*{20}c} {} & {c_{2} } & {9,10} \\ \end{array} } \\ {\begin{array}{*{20}c} {} & \vdots \\ \end{array} \begin{array}{*{20}c} {} & \vdots \\ \end{array} } \\ {\begin{array}{*{20}c} {} & {c_{27} } & {9,10} \\ \end{array} } \\ \end{array} } \right]$$

Similarly, the classical domain $R_{2}$, $R_{3}$, $R_{4}$ and $R_{5}$ of each level can be obtained.

2. Section domain $$R_{0}$$
  $$R_{0} = \left[ {\begin{array}{*{20}c} {\begin{array}{*{20}c} O & {c_{1} } & {0,10} \\ \end{array} } \\ {\begin{array}{*{20}c} {} & {c_{2} } & {0,10} \\ \end{array} } \\ {\begin{array}{*{20}c} {} & \vdots \\ \end{array} \begin{array}{*{20}c} {} & \vdots \\ \end{array} } \\ {\begin{array}{*{20}c} {} & {c_{27} } & {0,10} \\ \end{array} } \\ \end{array} } \right]$$
3. Ten people were invited to score the project according to its basic characteristics, with a scale of 1–100, where [90,100) is I excellent, [80, 90) is II good, [70, 80) is III moderate, [60, 70) is IV qualified, [0, 60] is V unqualified. The maximum and minimum values of the experts’ scores were removed to calculate the average, and the specific value $R_{x}$ of the elements to be evaluated was obtained, as shown in Table 5.

**Table 5:** Elements to be evaluated.

Weight	Expert 1	Expert 2	Expert 3	Expert 4	Expert 5	Expert 6	Expert 7	Expert 8	Expert 9	Expert 10	$R_{x}$
S_111_	90	86	86	93	94	89	94	95	86	86	89.75
S_112_	88	87	91	82	88	87	82	94	90	93	88.25
S_113_	60	67	64	70	70	55	65	67	74	56	64.88
S_114_	62	67	72	63	60	70	67	69	60	62	65.00
S_121_	82	81	90	90	80	78	86	78	80	86	82.88
S_122_	77	78	76	78	79	79	76	81	79	78	78.00
S_123_	59	55	55	60	57	64	66	55	64	58	59.00
S_124_	89	91	79	90	81	92	92	83	92	86	88.00
S_131_	78	82	78	77	75	82	77	81	81	84	79.50
S_132_	59	51	64	54	41	42	44	60	55	41	50.75
S_133_	86	96	86	96	87	85	92	97	86	92	90.13
P_211_	88	93	82	90	87	80	89	90	88	85	87.38
P_212_	84	81	87	86	84	88	84	87	92	89	86.13
P_213_	85	81	92	86	91	90	86	81	90	85	86.75
P_214_	90	88	76	84	77	81	90	77	87	76	82.50
P_221_	87	92	91	91	89	92	86	85	87	85	88.50
P_222_	89	82	86	78	80	88	81	83	83	76	82.63
P_223_	83	83	84	81	81	89	89	85	88	86	84.88
P_224_	75	77	82	81	73	70	77	79	73	72	75.88
P_231_	83	91	81	83	83	90	92	80	84	82	84.63
P_232_	84	86	88	84	82	86	81	90	86	83	84.88
P_241_	76	76	70	69	73	79	76	77	77	73	74.75
P_242_	85	89	86	87	87	87	83	84	84	88	86.00
O_311_	84	85	87	84	87	87	85	86	83	82	85.13
O_312_	76	84	85	84	82	82	79	83	78	81	81.63
O_321_	88	91	87	87	83	90	86	81	86	89	87.00
O_322_	85	86	93	88	94	89	85	93	95	90	89.75

### Correlation calculation

Taking the three-level index S_111_ as an example, the correlation was found by combining Eqs. (7) and (8):

$$K_{1} (c_{1} ) = 0.3500;\;K_{2} (c_{1} ) = - \;0.6500;\;K_{3} (c_{1} ) = - \;1.6500;\;K_{4} (c_{1} ) = - \;2.6500;\;K_{5} (c_{1} ) = - \;0.6083.$$

It can be seen from $K_{j0} (c_{1} ) = \mathop {\max }\nolimits_{{j \in \left( {1,2, \cdots ,m} \right)}} K_{j} (c_{1} )$ that the brownfield location condition S_111_ is Level I excellent. Similarly, the correlation of all indexes at the three levels can be calculated, as shown in Table 6.

**Table 6:** Correlation of three-level indexes.

Three-level index	Correlation					Quality level
	$K_{1} (x_{i} )$	$K_{2} (x_{i} )$	$K_{3} (x_{i} )$	$K_{4} (x_{i} )$	$K_{5} (x_{i} )$	
S_111_	− 0.0250	**0.0250**	− 0.9750	− 1.9750	− 0.4958	II
S_112_	− 0.1750	**0.1750**	− 0.8250	− 1.8250	− 0.4708	II
S_113_	− 2.5125	− 1.5125	− 0.5125	**0.4875**	− 0.0813	IV
S_114_	− 2.5000	− 1.5000	− 0.5000	**0.5000**	− 0.0833	IV
S_121_	− 0.7125	**0.2875**	− 0.2875	− 1.2875	− 0.3813	II
S_122_	− 1.2000	− 0.2000	**0.2000**	− 0.8000	− 0.3000	III
S_123_	− 3.1000	− 2.1000	− 1.1000	− 0.1000	**0.0167**	V
S_124_	− 0.2000	**0.2000**	− 0.8000	− 1.8000	− 0.4667	II
S_131_	− 1.0500	− 0.0500	**0.0500**	− 0.9500	− 0.3250	III
S_132_	− 3.9250	− 2.9250	− 1.9250	− 0.9250	**0.1542**	V
S_133_	**0.0125**	− 0.0125	− 1.0125	− 2.0125	− 0.5021	I
P_211_	− 0.2625	**0.2625**	− 0.7375	− 1.7375	− 0.4563	II
P_212_	− 0.3875	**0.3875**	− 0.6125	− 1.6125	− 0.4354	II
P_213_	− 0.3250	**0.3250**	− 0.6750	− 1.6750	− 0.4458	II
P_214_	− 0.7500	**0.2500**	− 0.2500	− 1.2500	− 0.3750	II
P_221_	− 0.1500	**0.1500**	− 0.8500	− 1.8500	− 0.4750	II
P_222_	− 0.7375	**0.2625**	− 0.2625	− 1.2625	− 0.3771	II
P_223_	− 0.5125	**0.4875**	− 0.4875	− 1.4875	− 0.4146	II
P_224_	− 1.4125	− 0.4125	**0.4125**	− 0.5875	− 0.2646	III
P_231_	− 0.5375	**0.4625**	− 0.4625	− 1.4625	− 0.4104	II
P_232_	− 0.5125	**0.4875**	− 0.4875	− 1.1500	− 0.4146	II
P_241_	− 1.5250	− 0.5250	**0.4750**	0.1750	− 0.2458	III
P_242_	− 0.4000	**0.4000**	− 0.6000	0.1750	− 0.4333	II
O_311_	− 0.4875	**0.4875**	− 0.5125	− 1.0750	− 0.4188	II
O_312_	− 0.8375	**0.1625**	− 0.1625	− 0.8250	− 0.3604	II
O_321_	− 0.3000	**0.3000**	− 0.7000	− 1.9000	− 0.4500	II
O_322_	− 0.0250	**0.0250**	− 0.9750	− 1.7000	− 0.4958	II

Significant values are in [bold].

### Comprehensive evaluation

The correlation of primary indexes and secondary indexes was calculated by Eq. (9) and $K_{j0} (c_{1} ) = \mathop {\max }\nolimits_{{j \in \left( {1,2, \ldots ,m} \right)}} K_{j} (c_{1} )$, as shown in Tables 7 and 8. Then, the integrated correlation of brownfields in the overall development quality evaluation was calculated:

$$K(O) = \left[ { - \;0.8087,\; - \;0.0729,\; - \;0.5647,\; - \;1.3289,\; - \;0.3652} \right]$$

**Table 7:** Correlation of secondary indexes.

Secondary index	Correlation					Quality level
	$K_{1} (x_{i} )$	$K_{2} (x_{i} )$	$K_{3} (x_{i} )$	$K_{4} (x_{i} )$	$K_{5} (x_{i} )$	
S_11_	− 1.2598	− 0.6799	− 0.7129	− 0.7460	**− 0.2900**	V
S_12_	− 1.3217	− 0.4862	− 0.5555	− 1.0073	**− 0.2797**	V
S_13_	− 1.6606	− 1.0071	− 0.9755	− 1.3008	**− 0.2232**	V
P_21_	− 0.4260	**0.3076**	− 0.5740	− 1.5740	− 0.4290	II
P_22_	− 0.6914	**0.1315**	− 0.3086	− 1.3086	− 0.3848	II
P_23_	− 0.5256	**0.4744**	− 0.4744	− 1.4744	− 0.4124	II
P_24_	− 0.9578	**− 0.0586**	− 0.0670	− 1.0422	− 0.3404	II
O_31_	− 0.6561	**0.3309**	− 0.3439	− 1.3439	− 0.3906	II
O_32_	− 0.1663	**0.1663**	− 0.8337	− 1.8337	− 0.4723	II

Significant values are in [bold].

**Table 8:** Correlation of primary indexes.

Primary index	Correlation					Quality level
	$K_{1} (x_{i} )$	$K_{2} (x_{i} )$	$K_{3} (x_{i} )$	$K_{4} (x_{i} )$	$K_{5} (x_{i} )$	
S_1_	− 1.4176	− 0.7352	− 0.7567	− 1.0173	**− 0.2637**	V
P_2_	− 0.6435	**0.2188**	− 0.3623	− 1.3565	− 0.3928	II
O_3_	− 0.4102	**0.2482**	− 0.5898	− 1.5898	− 0.4316	II

Significant values are in [bold].

### Evaluation results and analysis

According to the maximum affiliation principle $K_{j0} (O) = \mathop {\max }\nolimits_{{j \in \left( {1,2, \ldots ,m} \right)}} K_{j} (O_{j} ) = - \;0.0729$, the overall quality evaluation rating for the redevelopment of this brownfield project is II good, indicating that the site is suitable for development. The correlation of the primary indexes shows that the process quality and outcome indexes are both II good, but the quality of the structure indexes scores V unqualified.

All secondary indexes under the process index are II good, indicating that government policy support, process supervision and management, transparent and open information, technical guarantee from experts, and capital investment have significant advantages in the renovation project. It also shows that all participants strongly support the renovation project in the early stage.

All secondary indexes under the outcome index are also II good, indicating that the project has great benefits in both social and economic aspects and also revealing that residents are paying more and more attention to the environment and demanding a higher quality of life.

The geographic environments, human conditions and pollution conditions under the structural indexes are all evaluated as V unqualified. Specifically, the low evaluation of geographic environments is mainly caused by the low three-level index rating of use status and traffic conditions, which indicates that the current land use of the project is chaotic and unreasonable; traffic jam is severe despite the superior location. The low evaluation of human conditions is mainly caused by the low knowledge level of the three-level index of the brownfield, and the low evaluation of pollution conditions is mainly caused by the low knowledge level of the brownfield. The low assessment of the pollution condition is mainly caused by the large challenges in restoration technology. Therefore, we need to carefully consider the use and traffic planning of the renovated project. Transparency and openness in information dissemination are essential to raise public awareness of the hazards of brownfield sites and gain public support.

The secondary indexes of geographic environments, human conditions and pollution status under the structural indexes are all evaluated as V unqualified. Specifically, the low evaluation of geographic environments is mainly caused by the low three-level index rating of use status and traffic status, indicating that the current land use of the project is chaotic and unreasonable; traffic congestion is severe despite its superior location. The low evaluation of human conditions is mainly caused by the low environmental awareness of brownfield sites of three-level indexes. The low evaluation of pollution status is mainly caused by the large challenges in restoration technology. Therefore, the use and traffic planning of the renovated project should be considered carefully and comprehensively. Information should be transparent and open to raise public awareness of the hazards of brownfield sites and gain public support. In addition, technical efforts should be further concentrated to overcome difficulties and ensure adequate technical support for project implementation.

## Conclusion

With the expansion of urbanization, brownfield redevelopment projects in the process of urban renewal have become an important guarantee for green and sustainable urban development, alleviating the land tension for urban development and improving urban quality. Brownfield redevelopment is often challenged by complex issues such as environmental governance, the involvement of multiple stakeholders and high economic investment. Therefore, the main objective of this study is to form a scientific and reasonable ex-ante evaluation mechanism for brownfield redevelopment.

Firstly, in order to establish a reasonable evaluation index, this study introduces the structure-process-outcome theory, from the perspective of green sustainability, a comprehensive analysis of the basic conditions, practice principles and outcome orientation throughout the brownfield redevelopment is conducted from three dimensions: structure, process and outcome. The composition of influencing factors under each dimension is studied, and the composition of information elements under the interconnection of each dimension is analyzed. A complete evaluation index system of brownfield redevelopment is established by combining literature review and expert consultation, including three primary indexes, nine secondary indexes and 27 three-level indexes.

Secondly, to obtain a scientific computational model, the C-OWA operator assignment method, which is closely related to the decision data and has the good nature of normal distribution, is applied to the weight calculation of the index system of brownfield redevelopment. Its adoption eliminates the subjectivity of weight calculation and reflects the fairness of decision-making. The characteristics, quantitative values, objects and changes of the contradictory problems in brownfield redevelopment evaluation are effectively expressed by incorporating the topologic theory. The organic combination of the two enables scientific and reasonable modeling of brownfield redevelopment evaluation.

Finally, to validate the applicability of the evaluation mechanism. Brownfield redevelopment evaluation indexes and evaluation models are applied to Sanxiang Reconstruction Project. The evaluation results obtained that the total quality evaluation grade of the redevelopment of this brownfield project is Class II good, very suitable for development. However, the geographic environment, human conditions and pollution status of each secondary indicator evaluation are level V failed. Based on the results of the assessment, the following policy recommendations are made. First, effective planning should be done in the process of brownfield development, and brownfield development efforts should align with the surrounding environment. Second, the dissemination of brownfield knowledge in a transparent manner should be given priority to raise public awareness regarding the hazards of brownfield sites and to gain public support. The recommendation was fed back to the project decision makers and contributed well to the smooth running of the brownfield project.

It can be found through the above study that the brownfield redevelopment evaluation mechanism proposed in this paper has great practical significance, the use of the C-OWA operator-topology method can effectively reduce subjectivity in the evaluation process, but there remains a lack of clarity and uncertainty in the majority of the indicators. In the future, we will concentrate on optimizing the indicators and gathering more data to further increase the objectivity and plausibility of the evaluation results.

## Data Availability

All data used in this study are available from the corresponding author.
